# Reducing the prosthesis modulus by inclusion of an open space lattice improves osteogenic response in a sheep model of extraarticular defect

**DOI:** 10.3389/fbioe.2023.1301454

**Published:** 2023-12-07

**Authors:** Reza Sanaei, Charles Neil Pagel, Babatunde A. Ayodele, Bill Lozanovski, Thierry Beths, Martin Leary, Darpan Shidid, Endri Kastrati, Joe Elambasseril, Ulrich Bühner, Tom Williamson, Stewart Ryan, Milan Brandt

**Affiliations:** ^1^ Melbourne Veterinary School, Faculty of Science, The University of Melbourne, Parkville, VIC, Australia; ^2^ RMIT Centre for Additive Manufacturing, RMIT University, Carlton, VIC, Australia; ^3^ Stryker Australia Pty Ltd., St Leonards, NSW, Australia; ^4^ Stryker Leibinger GmbH & Co., KG, Freiburg, Germany

**Keywords:** stress shielding, endoprosthesis, osteointegration, Ti6Al4V-ELI, lattice, additive manufacturing, load bearing

## Abstract

**Introduction:** Stress shielding is a common complication following endoprosthetic reconstruction surgery. The resulting periprosthetic osteopenia often manifests as catastrophic fractures and can significantly limit future treatment options. It has been long known that bone plates with lower elastic moduli are key to reducing the risk of stress shielding in orthopedics. Inclusion of open space lattices in metal endoprostheses is believed to reduce the prosthesis modulus potentially improving stress shielding. However, no *in vivo* data is currently available to support this assumption in long bone reconstruction. This manuscript aims to address this hypothesis using a sheep model of extraarticular bone defect.

**Methods:** Initially, CT was used to create a virtual resection plan of the distal femoral metaphyses and to custom design endoprostheses specific to each femur. The endoprostheses comprised additively manufactured Ti6Al4V-ELI modules that either had a solid core with a modulus of ∼120 GPa (solid implant group) or an open space lattice core with unit cells that had a modulus of 3–6 GPa (lattice implant group). Osteotomies were performed using computer-assisted navigation followed by implantations. The periprosthetic, interfacial and interstitial regions of interest were evaluated by a combination of micro-CT, back-scattered scanning electron microscopy (BSEM), as well as epifluorescence and brightfield microscopy.

**Results:** In the periprosthetic region, mean pixel intensity (a proxy for tissue mineral density in BSEM) in the caudal cortex was found to be higher in the lattice implant group. This was complemented by BSEM derived porosity being lower in the lattice implant group in both caudal and cranial cortices. In the interfacial and interstitial regions, most pronounced differences were observed in the axial interfacial perimeter where the solid implant group had greater bone coverage. In contrast, the lattice group had a greater coverage in the cranial interfacial region.

**Conclusion:** Our findings suggest that reducing the prosthesis modulus by inclusion of an open-space lattice in its design has a positive effect on bone material and morphological parameters particularly within the periprosthetic regions. Improved mechanics appears to also have a measurable effect on the interfacial osteogenic response and osteointegration.

## 1 Introduction

Endoprosthetic reconstruction is one of the cornerstones of limb salvage surgery requiring extensive resection of bone. Current indications include osseous or soft-tissue tumors encroaching on bone, periprosthetic infections as well as the surgical management of arthritic and trauma patients (Hennessy et al., 2020). Based on current projections, by 2040, annual hip and knee replacement surgeries, in the US alone, are predicted to reach over 1.4 and 3.4 million respectively (Singh et al., 2019). Endoprostheses are commonly affected by complications such as mechanical and soft-tissue failure, periprosthetic infection and fractures as well as aseptic loosening, which may lead to subsequent revisions or limb amputation (Abu El Afieh et al., 2022). While the rate of complications associated with endoprostheses is not dissimilar to alternatives such as allogenic reconstructions (Albergo et al., 2017), it is beneficial to explore the possibility of limiting these adverse events. This is paramount especially when considering patients requiring chemo- and/or radiotherapeutic treatments who are at a higher risk of such complications (Novikov et al., 2019; Fujiwara et al., 2020; Abu El Afieh et al., 2022).

Early data suggests that altering the implant design to introduce porosity is the key to alleviating some of the risk (Ji et al., 2020; Guder et al., 2021). Effective incorporation of a surface lattice can improve post-operative soft-tissue and bone integration reducing surgical dead space and thus the risk of infection (Chen et al., 2009; Guder et al., 2021). The resulting improved soft tissue integration is thought to be associated with improved function and a reduced risk of soft tissue failure (Chen et al., 2009; Guder et al., 2021).

It has been suggested previously that titanium implants that incorporate open space lattice architectures in their design, have favorable osteoconductive properties, yielding excellent osteointegration when used in the reconstruction of long bones (Li et al., 2016; Crovace et al., 2020). Good quality osteointegration improves load sharing with the periprosthetic bone protecting the device from mechanical failure and premature loosening (Mavrogenis et al., 2009; Apostu et al., 2018; Ji et al., 2020). Moreover, the engineered porosity (if substantial) reduces device’s elastic modulus, which has been suggested to result in enhanced osteogenesis and reduced stress shielding of the periprosthetic bone (Huiskes et al., 1992; Sumner and Galante, 1992; Nouri and Hodgson, 2010; Moghaddam et al., 2016; Caffrey et al., 2018). Stress shielding is a common complication of endoprosthetic reconstruction surgery and is significant not only because of associated surgical failure, but also due to the resulting periprosthetic osteopenia, which significantly limits future treatment options (Nagels et al., 2003; Tagliero et al., 2020; Braig et al., 2021; Cho et al., 2021; Bendich et al., 2022). Selecting an appropriate prosthesis modulus is crucial to achieving the best possible functional and long-term outcomes for patients with medical implants because it ensures that the mechanical properties of the artificial implant closely match those of the surrounding natural tissues and bones, preventing issues like stress shielding and subsequent implant loosening, or implant failure that may arise if the prosthesis is too rigid or too flexible compared to the natural tissue.

Notwithstanding this rationale, many legislative agencies around the world still do not allow for routine incorporation of lattices into implants due to a dearth of *in vivo* and clinical data on their effectiveness and safety. Thus, further research is required to establish the therapeutic profile and safety of this approach in the design and manufacturing of endoprostheses. This work was undertaken to test the hypothesis that reducing the prosthesis modulus by incorporating a three-dimensional (3D) open-space lattice into its core structure, will improve host osteogenic response in load bearing locations and prevent stress shielding of the periprosthetic bone. To our knowledge, there are no previous studies that have directly investigated this hypothesis using a controlled *in vivo* model in a long bone.

Here, we describe our findings following experimental reconstruction of distal femoral extraarticular defects in a sheep model where two distinct structural designs of different moduli are compared. A sheep model was selected due to the relatively large size of bones in sheep and thus similar biomechanics to humans. To overcome the challenges associated with surgical planning and fitting of highly customized and geometrically complex endoprostheses, bone resections were performed using a robot-assisted approach followed by manual fitting of the prostheses. Our research questions were:

(1) Is a reduction in prosthesis modulus associated with an improved quantity and quality of periprosthetic cortical bone?
(2) Is a reduction in prosthesis modulus associated with an improved osteointegration, i.e., quantity and quality of interfacial and interstitial bone?

## 2 Materials and methods

### 2.1 Animals

A total of nine 2-year-old castrated male Merino sheep (42–56 kg body weight on arrival) were used for this study which were housed and maintained in the animal house facility of the Melbourne Veterinary School, The University of Melbourne, Victoria, Australia. Sheep were kept in groups of maximum 4 animals per pen and provided with a once-a-day ratio of pellets. Dry hay and water were provided *ad libitum*. An acclimatization period of at least 2 weeks was observed before subjecting the animals to any procedures. Four animals were randomly assigned to each of the two groups receiving either the solid- or lattice-core prostheses. Each holding pen contained a mix of sheep from both experimental groups. One of the animals in the lattice group was euthanized early on due to the incidence of a spiral fracture distal to the implant during the recovery period and had to be replaced by an additional animal (hence nine animals were used in total). The use of all animals in this study was approved by the Animal Ethics Committee of the Faculty of Veterinary and Agricultural Sciences, the University of Melbourne (Infonetica # 10442). All work was conducted in compliance with the Australian Code for the Care and Use of Animals for Scientific Purposes (2013).

Each animal was anaesthetized twice, once at the time of the initial CT scans for planning purposes and once at the time of surgery. All intravenous drugs and fluids were administered via an 18G over-the-needle catheter placed in a cephalic vein. For the surgeries, following an intravenous administration of midazolam (0.3 mg/kg), general anesthesia was induced using an intravenous bolus of propofol (4–6 mg/kg). A prophylactic antibiotic (cefazolin, 20 mg/kg IV) was administered at this time and repeated every 90 min thereafter. This was followed by IM administration of procaine penicillin G (15 mg/kg once daily) for 3 postoperative days.

Anesthetic maintenance was provided with isoflurane in oxygen (20–50 mL/kg/min) via an endotracheal tube. Intraoperative analgesia consisted of intravenous methadone (0.2 mg/kg) every 4 h, a constant rate infusion (CRI) of ketamine (10 mg/kg/min) and epidural morphine (0.1 mg/kg). The CRI was preceded by a bolus of ketamine (1 mg/kg, IV) at the time of surgical site preparation. The first dose of methadone was also given at this stage. At the end of the procedure and before turning off the isoflurane, a subcutaneous dose of meloxicam (1 mg/kg) was administered. A fentanyl patch (0.2 mg/kg/h) was placed on the antebrachium at this time and kept for 72 h. Following recoveries, animals were individually housed in a divided pen for 5 days (2 animals per pen) before being returned to the flock. Further postoperative pain management was provided by buprenorphine (0.01 mg/kg IM q12 h) and meloxicam (1 mg/kg PO q24 h) for a total of 5 days. Sheep were provided with a rubber matting postoperatively for the duration of the study.

The anesthesia protocol for the planning CT scans were similar to the surgeries, but methadone was replaced with butorphanol (0.2 mg/kg IV) with no further intraprocedural or postprocedural analgesia. All animals were subjected to intravital fluorochrome labelling of bone using alizarin red (25 mg/kg IV) and calcein (10 mg/kg IV), 20 and 5 days respectively before being euthanized at 12 weeks. Euthanasia was performed by a lethal IV administration of pentobarbital.

### 2.2 Imaging and pre-planning

For each animal, computed tomography (CT) using a Siemens Somatom Emotion 16 (Siemens Healthineers, Germany) was used to scan the right hind limbs. The CT images were then used to create a representative 3D map of the distal femur in a custom surgical planning software based on the MITK framework (DKFZ, Germany). A “reference” plan was created based on an average femoral bone geometry (lateral aspect), on which a curved osteotomy geometry was defined. For each sheep, the generated femur geometry was aligned to the reference plan, as to create a curvilinear lateral metaphyseal defect that was identical in all 9 femora (one sheep was excluded from the study due to a postoperative fracture).

### 2.3 The design of the endoprostheses

**FIGURE 1:**
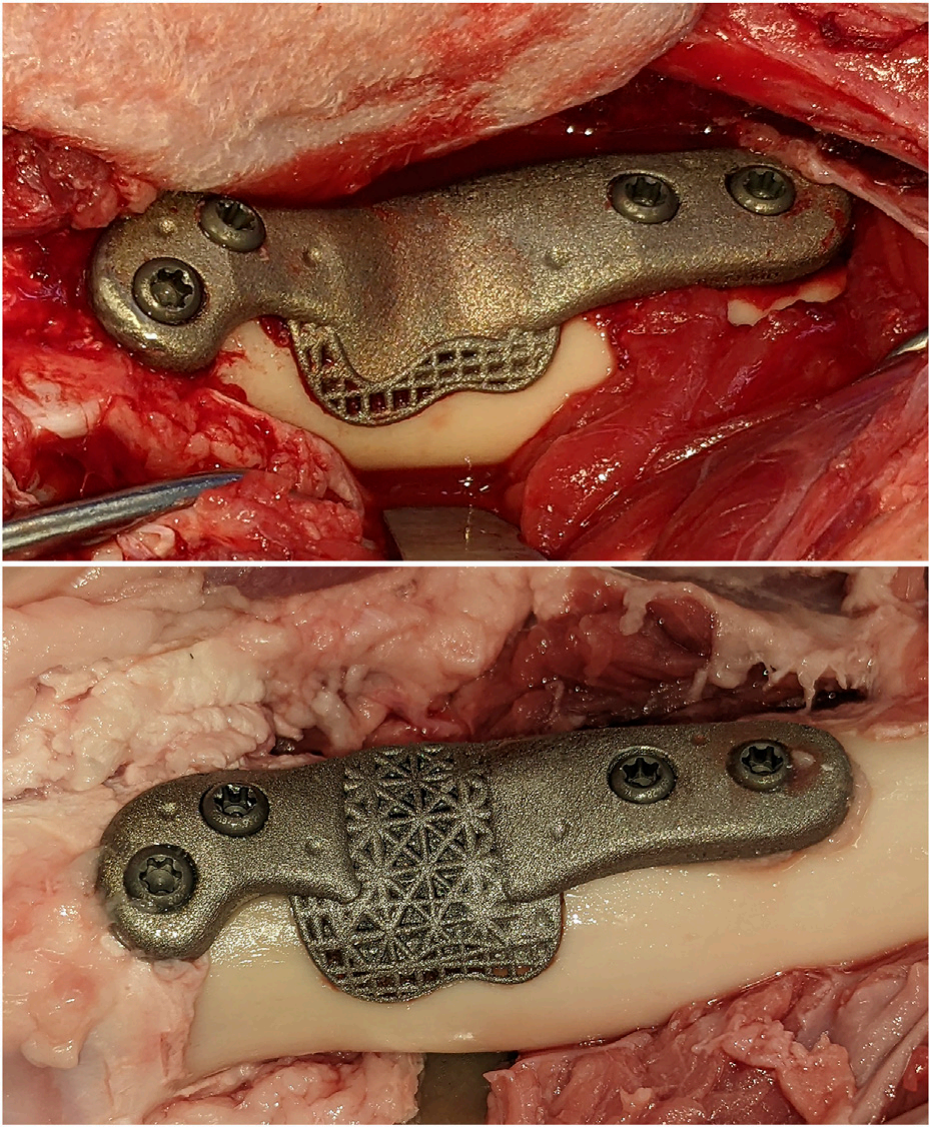
The solid and open-lattice prostheses within the surgically created defects. An identical interfacial lattice was incorporated into the bone facing aspects of both prosthesis designs.

Two types of endoprostheses were used in this study. Both designs had an identical convex bone interface matching the curvature of the pre-planned osteotomies. The curvature of the interface tapered into a proximal and distal flange each accommodating two fixation screws (total of 4 screws) resembling a bone plate with an expanded middle segment (Figure 1). The implants were designed to either have a solid core with a unit cell size of 2 mm as interfacial lattice (which faced the bone defect) or a lattice core within an identical interfacial lattice. The core lattice consisted of a gradient face-centered cubic with z-strut (FCCZ) lattice structure with a unit cell size of 4 × 4 × 4 mm. The surface-intersecting unit cells were modified to fit the geometric boundaries of the core. Strut diameter was linearly graded (0.7 mm–0.325 mm) as to avoid strut distortion from an excessive thermal gradient at the points of their connection with the flanges and to avoid stress concentrations when under loading. The interfacial lattice comprised a FCCZ lattice structure with a unit cell size of 2 × 2 × 2 mm. The strut diameter had a similar gradient (0.7 mm–0.325 mm in diameter). The solid core was estimated to have a 120 GPa modulus whereas the unit cells of the lattices had a 3–6 GPa modulus. For reference, the modulus of the ovine femur is about 22 GPa (Erickson et al., 2002).

Each endoprosthesis was additively manufactured out of Ti6Al4V-ELI (grade 23 titanium) powder in a SLM^®^125 machine (SLM Solutions, Germany) using a layer thickness of 30µm, scan speed of 375 mm/s, laser power of 100 W and a hatch distance of 130 µm. Following the manual removal of all support beams and a light deburring, implants were cleaned by dry-ice blasting (60 s per side). Further cleaning was performed using three 1-h cycles of ultrasonic cleaning in deionized water at 80°C and a diluted alkaline cleaning solution (Micro-90^®^, Cole-Parmer^®^, Illinois, United States). Each cycle was followed by a rinse in deionized water and submersion in a fresh batch of water and detergent. A fourth and final 1-h cycle in deionized water concluded the post-processing of implants.

### 2.4 Robotic resection and implantation

Surgical access to the distal femur was achieved via a lateral approach. To synchronize the pre-planned resection map with the actual bone, intra-operative registration was carried out to link four identifiable landmarks (medial and lateral condyles, trochlear groove, and part way up the femoral shaft) with corresponding fiducial points in the virtual 3D map of the same animal using a custom software. This was followed by the surgeon “painting” the surface of the bone with 50–150 additional fiducial points for the iterative closest point (ICP)-based final registration. Following registration and robotic osteotomies, prostheses were manually fit into each defect. Two 3.5 mm cortical screws (Stryker AxSOS 3 3.5 mm cortex screw, self-tapping, Stryker, United States) were used proximally, and two 4 mm trabecular screws (Stryker AxSOS 3 4.0 mm cancellous screw, full thread self-tapping, Stryker, United States) were used distally to secure each implant (Figure 1). The surgical site was then irrigated and closed in multiple layers. We set and adhered to a 2-week time limit for the whole process, from the planning CT to implantations, to simulate the realistic requirements of a clinical setting.

### 2.5 Microcomputed tomography

**FIGURE 2:**
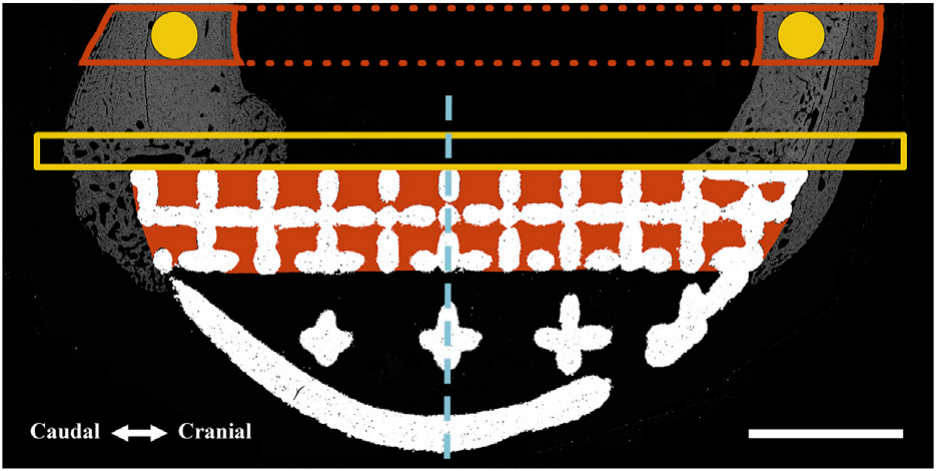
Regions of interest (ROIs) were defined for one-dimensional (perimeter) and two-dimensional (surface area and pixel density) measurements using an identical method for both BSEM (shown) and brightfield microscopy images. A similar method was used for volumetric analyses using micro-CT datasets (where measurements were performed). The interfacial ROI demarcated by the yellow box spanned the axial perimeter of the implant and extended for an extra 3 mm in both caudal and cranial directions and measured 1 mm in width. This region was further divided into cranial (Cr.Ax) and caudal (Cd.Ax) ROIs (dashed line depicts border). To take account of the periosteal reaction, the small caudal and cranial perimeters of the interfacial lattice were also analyzed; each started from the respective caudal or cranial end of the implant to the corresponding abaxial extent of the interfacial lattice. Area shaded in red depicts the interstitial ROI drawn by tracing the interior of the interfacial lattice. These ROIs were divided into cranial (Cr) and caudal (Cd) parts and were separately analyzed (dashed line depicts border). In the case of the full-lattice prostheses, a second interstitial ROI was defined by tracing the abaxial open lattice. The latter did not have an equivalent ROI in the solid prosthesis. The highlighted circles (1.5 mm in diameter) are periprosthetic ROIs (5 mm away from the prostheses) within the center of the original caudal and cranial cortices ignoring the periosteal and endosteal calluses. The full thickness of the caudal and cranial periprosthetic cortices were also traced (new cortex) and separately analyzed. Marrow diameter was measured within the area depicted by the dotted red lines. Scale bar equals 5 mm.

Following euthanasia at 12 weeks, femora were resected *en bloc* following perfusion fixation with 10% neutral buffered formalin (NBF) through a femoral artery catheter. Samples were defleshed as necessary, downsized and subjected to further fixation by submersion in 10% NBF for 7 days. Next, samples were washed using 3 changes of PBS while being agitated on a shaking plate (<60 RPM) for a total of 2 h. They were then placed in 50% ethanol at 4°C for 3 nights before being transferred to 70% ethanol. Micro-computed tomography (micro-CT) was performed using a Phoenix v|tome|x s240 CT system (GE Research, New York, USA) at an isometric resolution of 34.5 µm/voxel, with a peak voltage of 180 kV, current of 130 μA, integration time of 500 m, frame averaging of 3, image skip of 2, and a copper filter of 0.1 mm to reduce beam hardening. Datasets were rotated and cropped based on consistent landmarks in DataViewer version 1.5.6.2 64-bit (Bruker Micro CT, Aartselaar, Belgium) before being imported into CT Analyzer software version 1.13.11.0 (Bruker Micro CT, Aartselaar, Belgium Bruker, RRID:SCR_021338) for analysis. Volumes of interest (VOIs) were defined within the caudal and cranial periprosthetic cortices using similar criteria as those shown for histomorphometry in Figure 2. Due to the presence of metal related artefacts, the interfacial and interstitial VOIs were deemed unsuitable for quantitative analysis.

### 2.6 Histological processing

Following micro-CT, dehydration was carried out at 4°C using graded concentrations of ethanol with 15 min of vacuum applied after each change to remove trapped air bubbles. Dehydration took a total of 5 weeks. Clearing was performed using two changes of toluene for a total of 7 days. Samples were embedded using a modified method previously described (Emmanual et al., 1987). Briefly, cleared samples were infiltrated with the thin methyl methacrylate (MMA) solution (destabilized MMA + 1% dibutyl phthalate + 0.05% [w/v] benzoyl peroxide) for 3 weeks followed by the thick MMA solution (destabilized MMA + 1% dibutyl phthalate + 3% [w/v] benzoyl peroxide) before being polymerized at 15°C. Polymerized blocks were incubated overnight at 35°C and 40°C on separate days before further processing.

Embedded samples were orientated and trimmed as necessary to allow reproducible identification of sample landmarks and the proximal and distal limits of the implants. Further trimming was performed to remove the medial cortices. Blocks were marked at 2 mm intervals starting proximally at the level of the first screw closest to the center (histological section 1) all the way to the level of the corresponding screw in the distal flange (histological section 9). Blocks were glued to chucks and cut perpendicular to the long axis of bone using an IsoMet low speed saw (Buehler, Illinois Tool Works Inc., IL, United States) fitted with an IsoMet precision blade (IsoMet Blade, 15HC, 127 mm). Sections selected for brightfield, and fluorescence microscopy (histological sections 3, 5, 8) were glued to plexiglass and ground to a 50 µm thickness on a series of silicone carbide sandpapers and diamond suspensions using a custom-built planar microgrinder (Sanaei, 2023). Prepared sections were polished using diamond suspensions on polishing cloths using the same setup. When backscattered electron microscopy (BSEM) was intended (histological sections 4, 6), 2 mm blocks were ground until planar followed by polishing and carbon coating.

### 2.7 Microscopy

Sections prepared for BSEM were coated with a 20 nm carbon layer under a high vacuum (Safematic CCU-010 HV, Safematic Switzerland). BSEM images were obtained using a backscattered detector and electron beam setting of 10 kV voltage and 20 nA current at 10 mm working distance and 300X magnification (FEI Teneo Volumescope, Thermo Fisher Scientific, Hillsboro, OR, United States). BSEM image tiles were stitched using MAPS (ThermoFisher Scientific, MA, United States, RRID:SCR_024446). Stitched image files were downsized eighty percent for further analysis.

To identify the fluorochrome labels (histological sections 5, 8), unstained sections were scanned using the ×10 objective lens in a ZEISS Axioscan 7 slide scanner (Carl Zeiss AG, Oberkochen, Germany). FITC and Cy3 filters were used to detect calcein and alizarin labels respectively. Brightfield microscopic images (histological sections 3, 5, 8) were obtained following Masson-Goldner staining using the ×10 objective lens in the same slide scanner.

### 2.8 Static histomorphometry

ImageJ (National Institutes of Health, Bethesda, MD, United States, RRID:SCR_003070) was used for morphometric analysis of prepared BSEM and brightfield images by first defining standardized regions of interest (ROIs) within the periprosthetic, interfacial (bordering the implant) and interstitial regions (within implant pores) as shown in Figure 2. Bone area fraction and porosity were measured following segmentation of bone tissue as previously described (Dempster et al., 2013). Pixel intensity of bone tissue within each ROI (BSEM) was measured following identification by segmentation and expressed as cortical density (Ct.Dn). The percentage of implant surfaces spanned by bone tissue was also determined for the axial (facing the long axis of bone) as well as caudal and cranial interfacial perimeters (Figure 2).

### 2.9 Dynamic histomorphometry

Dynamic histomorphometry using the fluorescent labels alizarin red (red) and calcein (green) was performed to determine the extent and rates of mineralization and bone formation in the interfacial and interstitial ROIs shown in Figure 2 using histological sections 5, 8. Fluorescent images from each of the regions of interest were imported into ImageJ. Random fields from each ROI were exported as Tiff files after merging red and green channels, which were read into OsteoMeasure System, Version 4.10 (OsteoMetrics, Decatur, GA, USA, RRID:SCR_024447). The total bone surface, along with the bone surfaces labelled with either alizarin red or calcein (single labelled surfaces; sLS) and the bone surfaces labelled with both alizarin red and calcein (double labelled surfaces; dLS) were traced for each ROI. The dynamic histomorphometric parameters were derived by the OsteoMeasure software from the primary indices using the standard ASBMR nomenclature (Dempster et al., 2013).

### 2.10 Statistics

All measurements were performed by blinded operators where possible. Data analysis was performed by a different group of authors than those who conceptualized, allocated animals or ran the experiments. SPSS Statistics version 28 (IBM Corp^©^, Armonk, NY, United States, RRID:SCR_016479) was used for all analyses. Graphs were prepared using the Python (RRID:SCR_008394) data visualization libraries, Matplotlib and Seaborn (Hunter, 2007; Waskom et al., 2017). For each variable, the group means were compared using an Independent Samples *t*-Test. No significance threshold was set due to the exploratory nature of this work, a small sample size (*n* = 4) and current recommendations (Leopold and Porcher, 2017; Amrhein et al., 2019; Vail and Avidan, 2022). For effect size, Hedge’s g is reported which has been automatically corrected for the small sample size bias by SPSS Statistics. An effect size < 0.2 was considered small, > 0.5 was considered moderate and > 0.8 was considered large. Standard Error of Mean and Standard Error of Difference have been presented in graphs and tables respectively.

## 3 Results

Other than the one sheep that sustained a fracture during the immediate postoperative period, the recoveries were without incident. All animals were fully weight bearing on the operated limb within 2–3 weeks of surgeries. All prostheses were highly stable and well-integrated into the host bone during histological preparations and downsizing. The periosteal callus was usually more pronounced in the caudal region except for one animal in the solid implant group where it was more pronounced in the cranial region. In one case (lattice implant group), an ossicle was found within the caudal region which might have been caused by the lodgment of bone chips and particles produced at the time of osteotomies (osteotomies were always accompanied and followed by irrigation).

### 3.1 Periprosthetic ROIs

#### 3.1.1 Micro-CT

**FIGURE 3:**
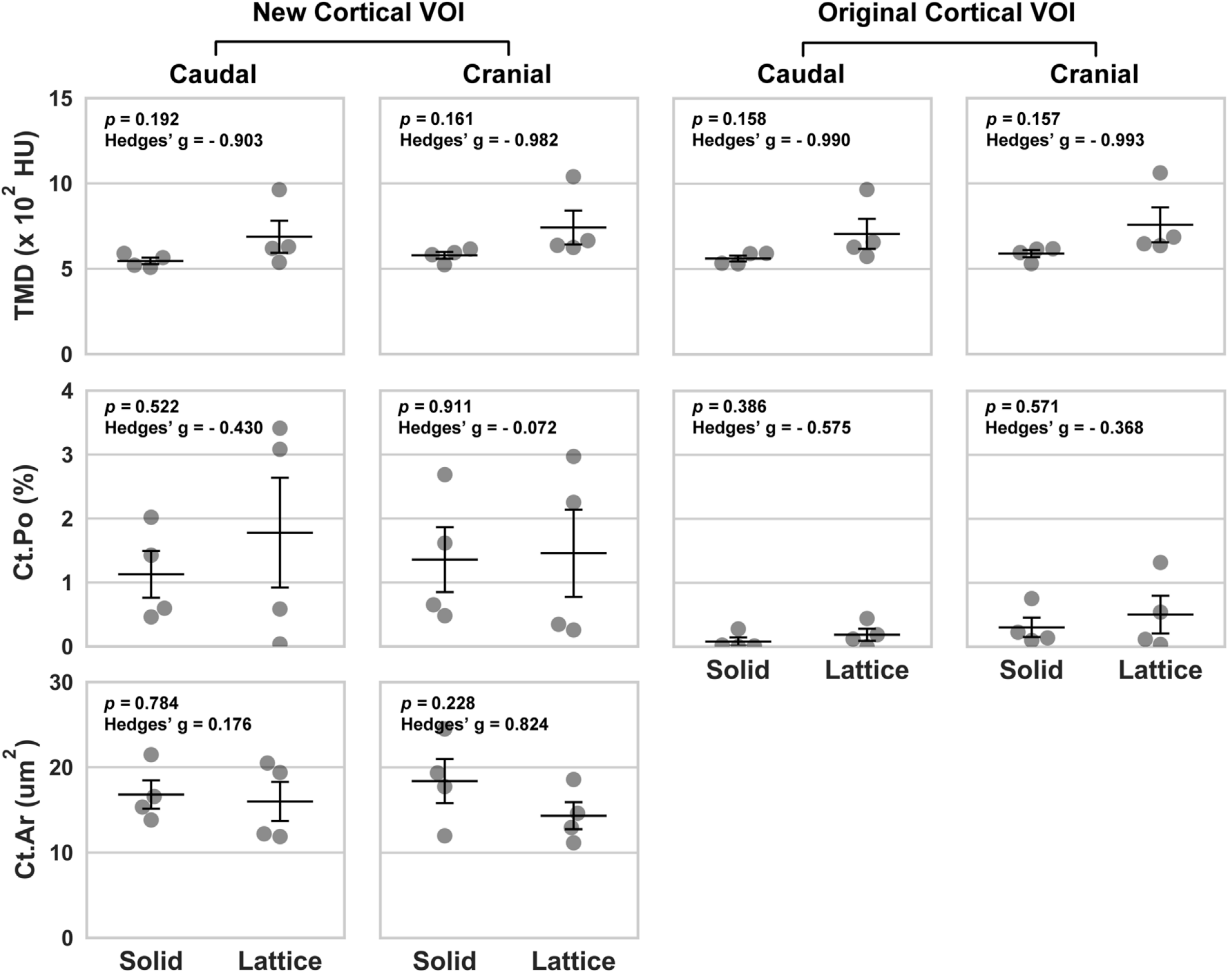
Volumetric data based on four periprosthetic VOIs following micro-CT imaging. The VOIs were defined using similar landmarks to those used for BSEM and brightfield analyses (Figure 2). Original cortical VOIs are a subset of the new cortical VOIs. Cortical area (Ct.Ar) was calculated by dividing the primary cortical bone volume values (Ct.V) by stack height (voxel size X number of slices per stack). As such, the presented Ct.Ar is a 3D driven index and is used as a proxy for the single dimensional cortical thickness (Bouxsein et al., 2010). TMD, tissue mineral density; Ct.Po, cortical porosity; *n* = 4; error bars represent SEM.

Data from the caudal and cranial periprosthetic VOIs is presented in Figure 3. The main differences observed between the two groups related to bone material density measurements (tissue mineral density; TMD). TMD appeared to be higher in the lattice group in all four caudal and cranial VOIs when compared to the control solid implant group. However, while similar between the two groups in the caudal VOI, cortical area (Ct.Ar) appeared to be lower in the cranial VOI of the lattice group.

#### 3.1.2 Back-scattered SEM

**FIGURE 4:**
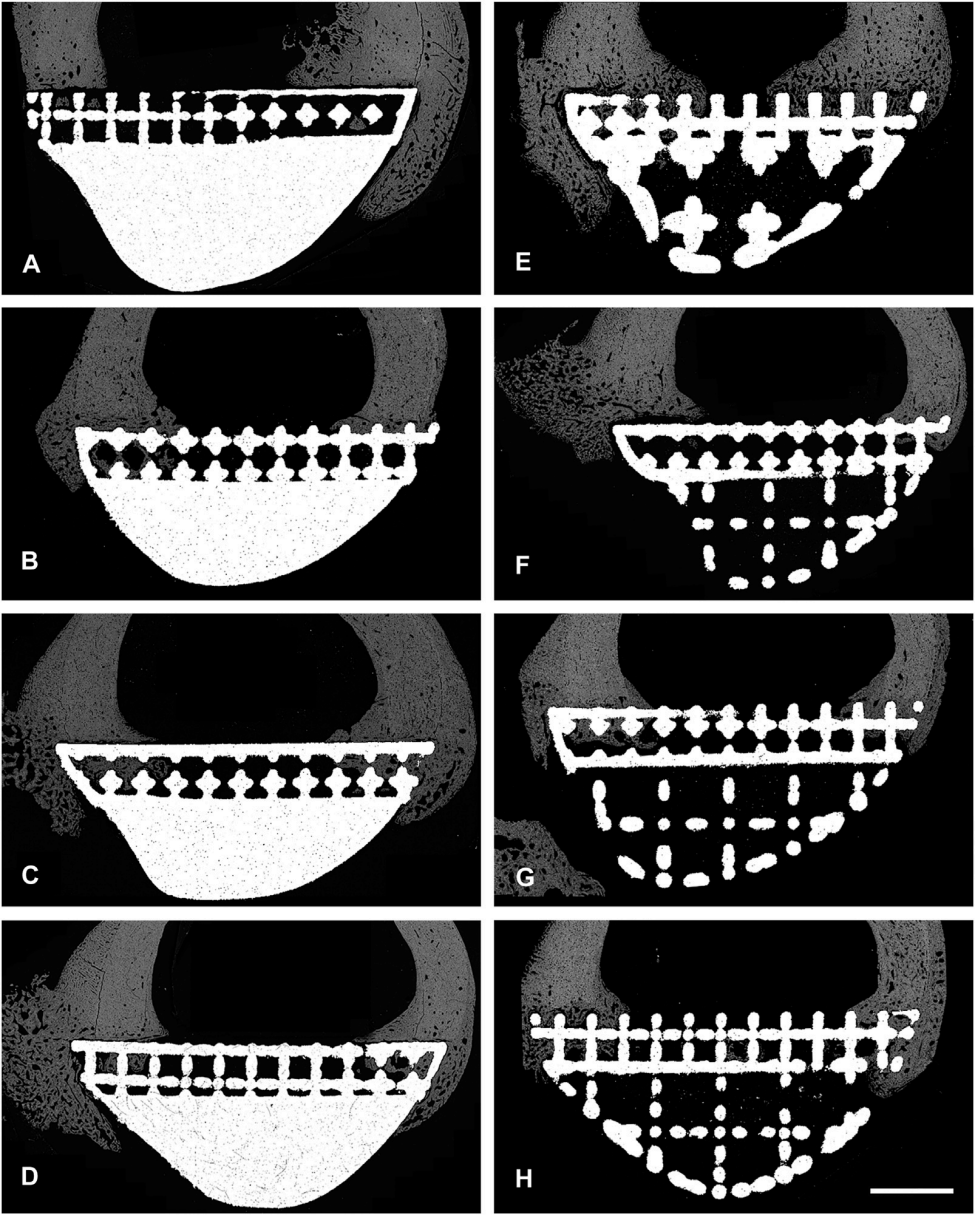
BSEM images from histological section 6. Grey signal indicates mineral. Cranial cortex is to the right side of each image. **(A–D)** solid implant group. **(E–H)** lattice implant group. Scale bar = 5 mm.

**FIGURE 5:**
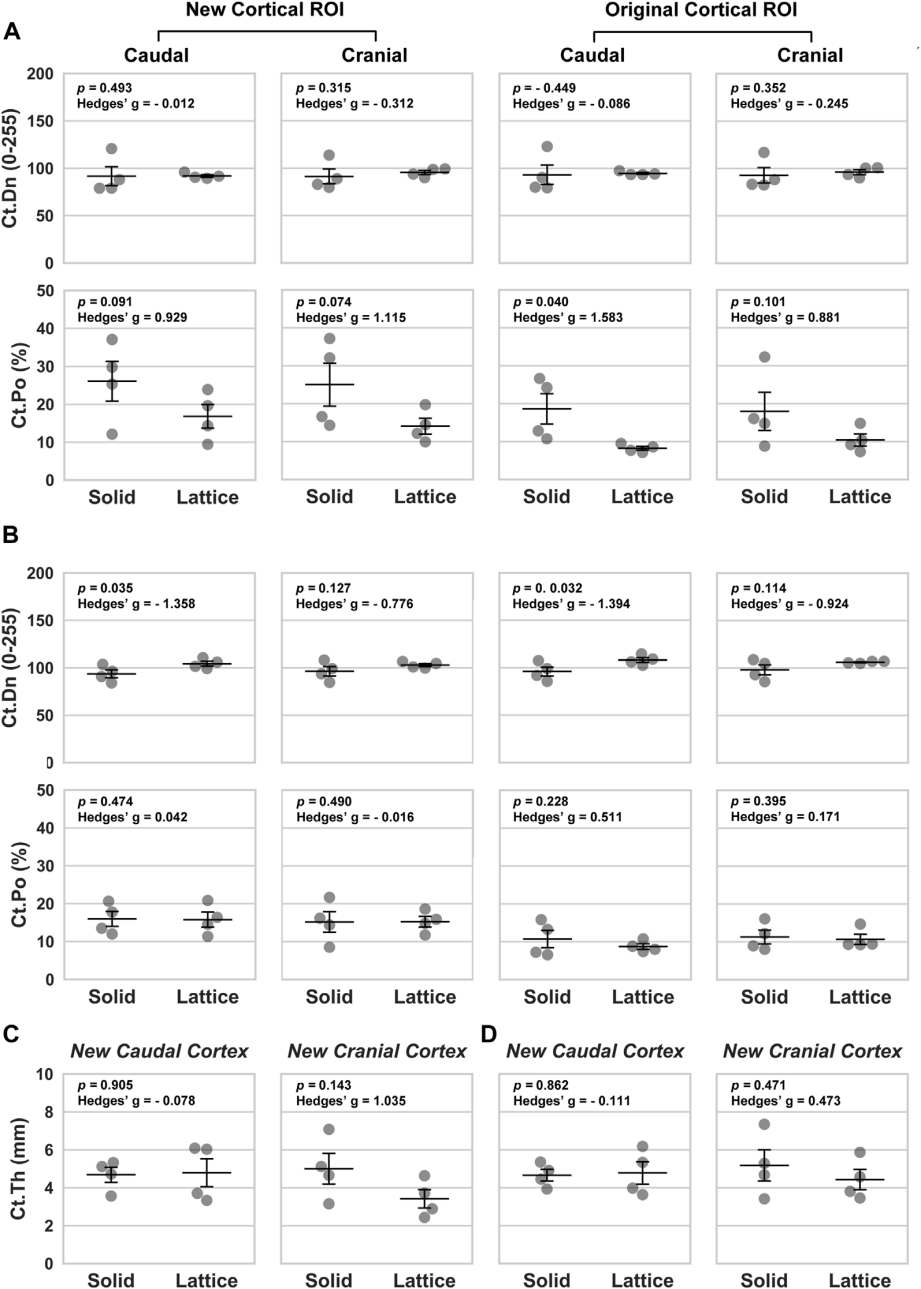
Morphological parameters based on four periprosthetic ROIs defined for BSEM images. **(A, C)** Data from histological section 4. **(B, D)** Data from histological section 6. Ct.Dn, cortical density; Ct.Po, cortical porosity; Ct.Th, cortical thickness. Data analyzed using a one-tailed Independent Samples *t*-Test; Hedges’ g values indicate effect size; *n* = 4; error bars represent SEM.

Back-scattered scanning electron microscopy (BSEM) was used to evaluate the periprosthetic cortical ROIs within histological section 4 (section 6 images shown in Figure 4). Analysis of pixel greyscale intensities (used as a proxy for cortical mineral density; Ct.Dn) indicated that here too, the lattice group had higher values in all 4 periprosthetic ROIs which was however only notable in histological section 6 (Figures 5A, B). Interestingly, cortical porosity (Ct.Po) values complemented this finding, being lower in the lattice group, this time, only in histological section 4 (Figure 5). Cortical thickness (Ct.Th), like micro-CT, appeared to be lower in the cranial cortex of the lattice implant group in histological section 4 with similar values in all other three ROIs.

### 3.2 Osteointegration: interfacial ROIs

**FIGURE 6:**
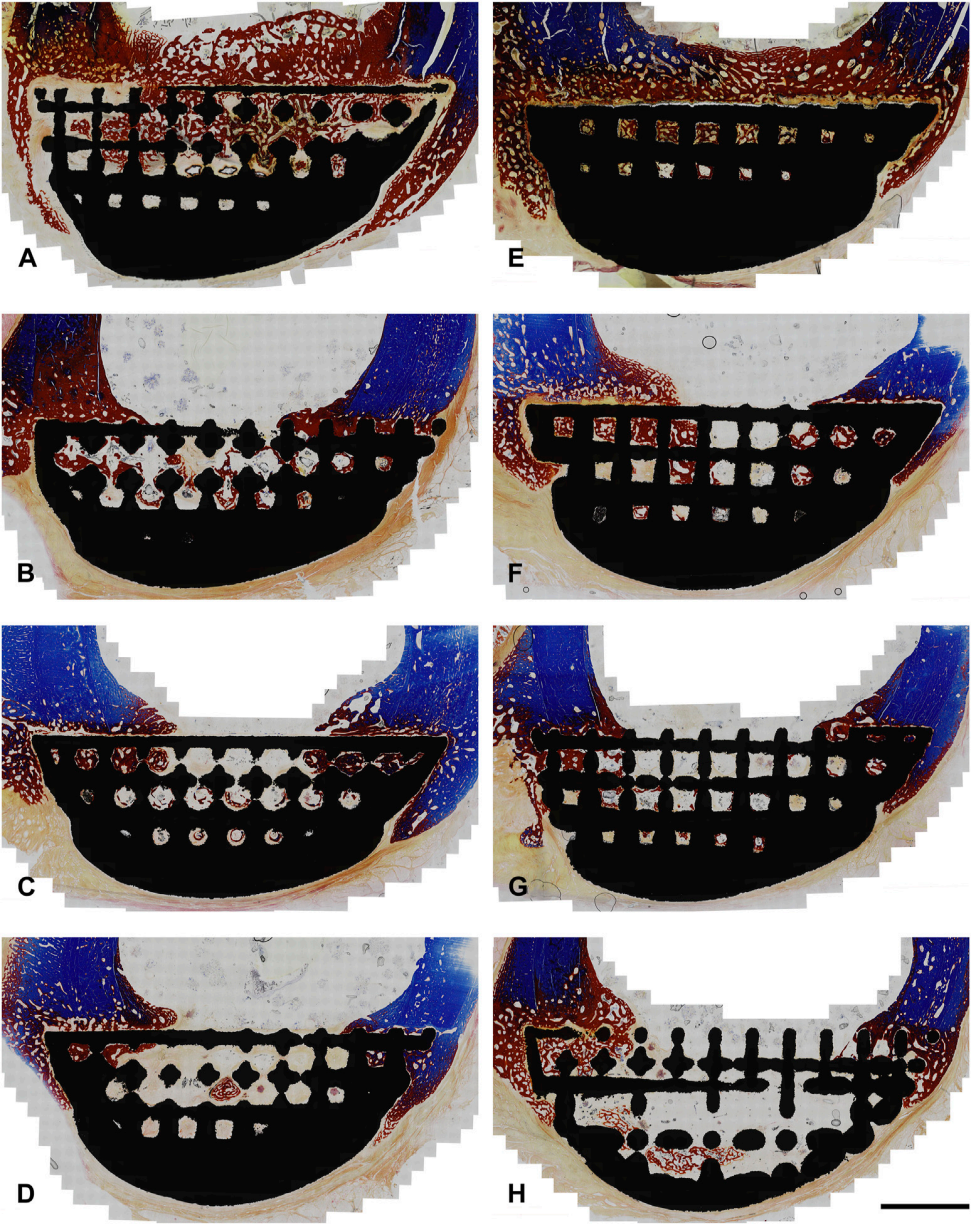
Photomicrographs from histological section 3 (Masson-Goldner Trichrome; original magnification ×10). Sections are from the boundary between the interfacial lattice and the implant core (lattice core is exposed in the bottom right image). Regions stained in blue are mineralized bone whereas regions stained in red are either osteoid or bone tissue that is partially mineralized. Cranial cortex is to the right side of each image. **(A–D)** solid implant group; **(E–H)** lattice implant group. Scale bar equals 5 mm.

To evaluate the effect of reduced modulus on osteointegration, interfacial ROIs (Figure 2) were histologically assessed and various area and perimeter as well as dynamic indices measured (Tables 1, 2). Static parameters were measured using BSEM images from histological sections 4 and 6 as well as Masson-Goldner-stained histological sections 3, 5 and 8 (section 3 images shown in Figure 6). Where BSEM was used, pixel intensity is also reported. Histological section 3 was only used for perimeter measurements. Osteoid measurements were only completed for histological section 5. Dynamic histomorphometry was completed for histological sections 5 and 8.

**TABLE 1:** Comparison of derived static interfacial parameters between the lattice and solid implant groups.

Index	Section/ROI	Two-tailed p	Hedges’ g^a^	Mean difference^a^	Std. Err of diff	Lower 95% CI of diff	Upper 95% CI of diff
B.Ar/T.Ar (%)	4/Cd.Ax	0.406	−0.55	−14.55	16.3	−54.43	25.32
	5/Cd.Ax	0.419	−0.53	−9.21	10.62	−35.19	16.77
	6/Cd.Ax	0.963	−0.03	−0.27	5.65	−14.09	13.55
	4/Cr.Ax	0.060	0.05	0.6	7.69	−18.21	19.4
	5/Cr.Ax	0.542	0.40	5.98	9.25	−16.66	28.61
	6/Cr.Ax	0.397	0.56	5.54	6.07	−9.32	20.4
	5/Cd.Ax	0.327	−0.66	−14.15	13.25	−46.58	18.27
	5/Cr.Ax	0.852	−0.12	−2.33	11.94	−31.54	26.88
Bone Density (0–255)	4/Cd.Ax	0.772	0.19	2.61	8.61	−18.45	23.67
	6/Cd.Ax	0.255	−0.77	−5.86	4.66	−17.25	5.54
	4/Cr.Ax	0.932	−0.06	−0.69	7.7	−19.54	18.16
	6/Cr.Ax	0.302	−0.69	−4.66	4.13	−14.75	5.44
Perimeter Spanned by Bone (%)	3/Ax	0.015	2.08	16.41	4.84	4.56	28.27
	4/Ax	0.685	0.26	5.74	13.46	−27.19	38.67
	5/Ax	0.840	−0.13	−2.81	13.35	−35.49	29.87
	6/Ax	0.711	0.24	5.08	13.06	−26.89	37.05
	8/Ax	0.296	0.703	22.24	19.43	−25.31	69.80
	3/Cd	0.222	−0.84	−31.22	22.90	−87.26	24.82
	4/Cd	0.834	−0.14	−11.46	51.11	−153.92	131.01
	5/Cd	0.214	−0.85	−35.90	25.86	−99.18	27.37
	6/Cd	0.599	0.34	20.27	36.54	−69.15	109.69
	8/Cd	0.391	0.61	3.52	3.52	−7.68	14.72
	3/Cr	0.599	−0.34	−20.86	37.61	−112.90	71.17
	4/Cr	0.581	0.36	32.55	55.81	−104.02	169.11
	5/Cr	0.904	−0.08	−4.14	33.02	−84.93	76.65
	6/Cr	0.785	−0.18	−21.12	73.92	−201.98	159.75
	8/Cr	0.037	−1.64	−64.65	24.18	−123.81	−5.48

^a^ Negative values indicate higher lattice values.

Ax, axial; Cd, caudal; Cr, cranial; Cd.Ax, caudal axial ROI; Cr.Ax, cranial axial ROI.

**TABLE 2:** Comparison of dynamic interfacial parameters between the lattice and solid implant groups.

Index	Section #	Two-tailed p	Hedges’ g^a^	Mean difference^a^	Std. Err of diff	Lower 95% CI of diff	Upper 95% CI of diff
sL/BS (%)	5	0.894	−0.08	−1.50	10.83	−28.01	25.01
	8	0.349	0.62	16.92	16.66	−23.86	57.69
dL/BS (%)	5	0.495	0.45	4.00	5.50	−9.46	17.46
	8	0.270	0.77	7.83	6.21	−8.75	24.41
MAR (µm/day)	5	0.157	−0.99	−0.58	0.36	−1.47	0.30
	8	0.42	−0.58	−9.54	10.12	−41.74	22.66
BFR (µm/day)	5	0.518	−0.42	−0.05	0.08	−0.24	0.13
	8	0.499	−0.47	−0.36	0.47	−1.84	1.12
BFR/BS (µm^3^/µm^2^/day)	5	0.794	−0.17	−0.00	0.01	−0.03	0.03
	8	0.526	−0.41	−0.02	0.03	−0.09	0.05

^a^ Negative values indicate higher lattice values.

Histology showed that the interfacial lattices in both implant groups were overall in contact with the host bone, marrow or fibrous tissue depending on location. Periosteal and endosteal callus was being remodeled and replaced with mature lamellar bone. A fibrous tissue capsule was found surrounding all abaxial surfaces which was continuous with the callus and occasionally continued onto the axial interface of the implants (facing the osteotomy defects). In many areas, evidence of intramembranous ossification could be seen at the interface of this layer and the newly formed bone. In some areas of the implant site, calcified cartilage was observed, indicating the presence of endochondral ossification in the newly formed bone. There was no evidence of inflammation or foreign body reaction in any of the sections.

While many indices were similar between the two groups, it was found that the axial interfacial perimeters were more thoroughly covered by bone in the solid implant group in histological sections 3 and 8 (Table 1). The cranial interfacial perimeters, on the other hand, were more extensively spanned by bone in the lattice implant group in histological section 8. Of the dynamic derived parameters, while mineral apposition rate (MAR) was higher in the lattice implant group, statistical analysis remained unequivocal (Table 2).

### 3.3 Osteointegration: interstitial ROIs

Static histomorphometry was performed for histological sections 4, 5 and 6 (Table 3). Dynamic histomorphometry was performed for histological sections 5 and 8 (Table 4). Our analyses did not point to any clear differences between the two groups here. Evidence of bony ingrowth into the core of the lattice implant group was apparent in 3 instances across 2 sections that were evaluated for this purpose. This could not be directly compared with the solid group for obvious reasons.

**TABLE 3:** Comparison of derived static interstitial parameters between the lattice and solid implant groups.

Index	Section/ROI	Two-tailed p	Hedges’ g^a^	Mean difference^a^	Std. Err of diff	Lower 95% CI of diff	Upper 95% CI of diff
B.Ar/T.Ar (%)	4/Cd	0.633	−0.31	−4.09	8.13	−24	15.81
	5/Cd	0.684	−0.26	−3.88	9.06	−26.05	18.29
	6/Cd	0.475	−0.47	−4.01	5.26	−16.89	8.87
	4/Cr	0.422	−0.53	−5.47	6.34	−20.98	10.05
	5/Cr	0.840	0.13	1.02	4.81	−10.75	12.78
	6/Cr	0.350	−0.62	−3.96	3.91	−13.54	5.61
O.Ar/B.Ar (%)	5/Cd	0.430	−0.56	−1.74	1.92	−7.80	4.32
	5/Cr	0.999	0.00	−0.01	10.56	−25.84	25.83
Bone Density (0–255)	4/Cd	0.608	0.33	3.75	6.95	−13.24	20.75
	6/Cd	0.284	−0.72	−3.52	2.99	−10.85	3.8
	4/Cr	0.960	0.03	0.33	6.35	−15.21	15.88
	6/Cr	0.660	−0.28	−1.53	3.31	−9.62	6.56

^a^ Negative values indicate higher lattice values.

Cd, caudal; Cr, cranial.

**TABLE 4:** Comparison of dynamic interstitial parameters between the lattice and solid implant groups.

Index	Section #	Two-tailed p	Hedges’ g^a^	Mean difference^a^	Std. Err of diff	Lower 95% CI of diff	Upper 95% CI of diff
sL/BS (%)	5	0.689	−0.26	−9.78	23.27	−66.72	47.15
	8	0.222	0.93	32.69	21.56	−34.02	99.40
dL/BS (%)	5	0.645	0.30	4.45	9.18	−18.02	26.91
	8	0.349	0.62	7.49	7.37	−10.55	25.53
MAR (µm/day)	5	0.626	−0.32	−0.65	1.24	−4.09	2.78
	8	0.222	0.84	1.82	1.33	−1.44	5.08
BFR (µm/day)	5	0.543	−0.40	−0.07	0.10	−0.36	0.27
	8	0.209	0.86	0.14	0.10	−0.11	0.40
BFR/BS (µm^3^/µm^2^/day)	5	0.599	−0.34	−0.01	0.01	−0.04	0.03
	8	0.273	0.74	0.01	0.01	−0.01	0.03

^a^ Negative values indicate higher lattice values.

## 4 Discussion

Our study reveals that that at the 12-week mark post-surgery, both open-lattice and solid core prostheses remain intact and are well integrated with the host tissues largely to the same extent. This points to a favorable characteristic of titanium lattices that when strategically incorporated into endoprostheses, can help minimize surgical dead space and improve both bone and soft tissue integration (research question 2). Notably, the results of our analyses suggest that modifying the prosthesis modulus of elasticity has a measurable effect on the periprosthetic and interfacial bone. The incorporation of an open lattice structure to minimize the implant modulus effectively reduces stress shielding of the surrounding bone, as predicted (research question 1).

Overall, the small sample size made statistical interpretations challenging. We have reported all *p* values in conjunction with the corresponding effect sizes (Hedge’s g) and have avoided flagging any results as statistically significant or otherwise as suggested by other groups (Leopold and Porcher, 2017; Amrhein et al., 2019; Vail and Avidan, 2022). We felt that this is appropriate due to the exploratory nature of this work and the small group size that would have led to a high risk of type II errors (Leopold and Porcher, 2017; Amrhein et al., 2019; Vail and Avidan, 2022). In this respect, the effect sizes should be paid special attention to. Given the early evaluations (12 weeks), and considering the reported effect sizes, these results are indeed promising.

### 4.1 The periprosthetic ROIs

Our micro-CT and BSEM evaluations suggest that the periprosthetic cortices in the lattice implant group had a higher mineral density/pixel intensity than the equivalent regions in the solid implant group. BSEM-derived porosity measurements complemented this finding, being lower in the lattice implant group. Micro-CT-derived porosity, nevertheless, did not appear to follow any meaningful patterns. To resolve this conflict, it must be noted that micro-CT of large metallic implants is frequently affected by photon starvation and beam hardening artefacts, which was also the case in our study so much so that we were unable to use our datasets for the purpose of interfacial and interstitial evaluations without risking significant error. BSEM, on the other hand, has far greater resolution and is not at all impacted by the presence of metals. As such, more weight is placed on BSEM-derived porosity measurements in interpreting the results. Specifically, in the caudal cortex, one of the two sections evaluated by BSEM showed markedly higher mean pixel intensity (a proxy for tissue mineral density) in the lattice implant group, indicating denser bone formation (*p* = 0.035, Hedges’ g = −1.358). Additionally, both caudal and cranial cortices in one of the two evaluated sections exhibited lower porosity in the lattice implant group (caudal: *p* = 0.091, Hedges’ g = 0.929; cranial: *p* = 0.074, Hedges’ g = 1.115). Furthermore, the central ROI within the caudal cortex, excluding the callus, also showed less porosity in the lattice implant group (*p* = 0.040, Hedges’ g = 1.583). These findings suggest that the lattice implant’s open structure may contribute to greater bone density and reduced porosity, potentially indicating improved bone integration and remodeling around the implant site.

The above-mentioned differences in pixel intensity and porosity support our hypothesis that an implant with a lattice architecture and thus lower modulus shares more load with the host bone and therefore stimulates the osteogenic response when compared to a similarly designed higher modulus solid implant. This notion is in accord with previous studies describing the effects of stress shielding associated with bone plates (Tonino et al., 1976; Moyen et al., 1978; Uhthoff and Finnegan, 1983; Uhthoff et al., 1993).

A perplexing aspect of our findings was the observed differences between the cranial and caudal VOIs/ROIs. Importantly, despite bone density and porosity differences supporting our hypothesis, the cranial periprosthetic Ct.Ar (micro-CT) and Ct.Th (BSEM–histological section 4) values were smaller in the lattice implant group in comparison to the control group. Previously, a biomechanical study by Woo et al. proposed that bone plate induced stress shielding manifests in the form of cortical thinning rather than diminished mechanical properties of the bone tissue *per se* (Woo et al., 1976). It must, however, be noted that based on current understanding, the latter characteristic is attributed to bone mineralization and bone porosity (Hart et al., 2017), both of which allude to the onset of a gradual bone loss in the solid implant group in our study. Contrary to the conclusion proposed by Woo et al., other experimental results confirm our opinion that porosity is a key variable that increases by stress shielding (Tonino et al., 1976; Moyen et al., 1978; Uhthoff and Finnegan, 1983; Uhthoff et al., 1993). Bone mineralization is also thought to respond to strain based on available data (Skedros et al., 1994; Isaksson et al., 2009).

Under physiological conditions, the disparities in loading conditions between different regions in bone often result in regional differences in cortical thickness, structural organization and/or mineralization (Skedros et al., 1994; Skedros et al., 2019). Of note, the natural curvature of the femur in this distal location results in the generation of eccentric axial compression and bending when loaded during normal weight bearing and locomotion exposing the caudal and cranial cortices to compressive and tensile strains respectively. The current observation that the cranial cortex was thinner in the lattice implant group than the solid implant group may be partially explained if one assumes a more pronounced host response to compressive than tensile strains. Zhong et al. have previously shown that *in vitro*, compressive strains are significantly more potent than tensile strains in stimulating Wnt signaling in osteoblasts (Zhong et al., 2013). Wnt signaling is a well-known pro-osteoblastic pathway that positively regulates bone formation and accrual (Kim et al., 2013).

That said, it has been suggested that in the sheep tibia, loading-induced regional changes in bone mass are not closely linked to local strain magnitude and as such inference of functional loading history from bone shape should be done judiciously (Wallace et al., 2014). It is also possible that the complex contour of the prostheses used here introduces a different strain distribution pattern to these assumptions. Callus buttressing is another factor that should be considered when interpreting these results. All femora evaluated had a more extensive callus caudally. It must be also noted that from the viewpoint of biomechanics, a sole reduction in cortical thickness or area can sometimes be associated with an increase in flexural and torsional rigidity (Uhthoff et al., 1993). As we did not complete a mechanical study, this remains to be determined by future investigations. Finite element analysis may also be useful to shed light on these findings.

### 4.2 Osteointegration

Evaluation of the interfacial ROIs seemed to indicate that osteointegration varied slightly across the interface; a better bone-prosthesis contact was observed in the axial perimeter for the solid implant group and in the cranial perimeter for the lattice implant group. No difference was observed between the two groups within the interstitial ROIs though the lattice core allowed for some bone ingrowth which could be considered an advantage of this design. While the difference in modulus and the resulting change in mechanics can help explain the subtle differences observed, the presence of an identical interfacial lattice in both prosthesis designs may be the key in understanding these findings. This interfacial lattice was included in both designs to take advantage of osteointegration and better load sharing in both groups hence a more relevant comparison. Variation of congruence at implant-bone interface is a well-known issue impacting the validity of research on the biomechanics of bone plate-type implants (Pilliar et al., 1979; Uhthoff et al., 1993). Having a similar interface across the groups also enabled us to assess the effect of modulus on osteointegration. This, however, has likely minimized the gap between the two groups as the interfacial lattice has a lower modulus than the solid core. Thus, a modulus gradient in the solid prosthesis is expected with lower modulus aspects being in direct contact with the host bone which can explain the observed overall similarities between the two groups in this region.

### 4.3 Limitations

Other than the small sample size, the similarity between the two implant designs, both having an identical interfacial lattice, can be considered a limiting factor in the current model as discussed before. Additionally, this study includes observations following a 12-week period. This is a short timeline considering the permanent nature of endoprostheses in an actual clinical setting. We based our 12-week time point for this study on the report that most of the loss in bone rigidity occurs within the first 8 weeks following plate buttressing with histological changes becoming more and more obvious in the subsequent weeks (Uhthoff et al., 1993). While invaluable insight is gained from this study, longer observations are required to thoroughly investigate our hypothesis.

### 4.4 Conclusion

The study demonstrates that reducing the prosthesis modulus by inclusion of a load-bearing open-space lattice in its design can effectively reduce stress shielding of the periprosthetic bone. This can potentially reduce the risk of periprosthetic osteopenia long term hence the risk of fractures. The modified biomechanical profile can also change the interfacial osteogenic response and the resulting osteointegration. This has significant implications for orthopedic implant design, suggesting that strategically incorporating load-bearing open-lattice structures into endoprostheses could enhance surgical outcomes and long-term stability by optimizing bone-implant interactions. Combined with the advantages of 3D computer aided design and additive manufacturing in patient customization and therefore the resulting improved bone fit, it is our opinion that use of lattices should be an indispensable part of many future applications of the technology in joint and bone reconstruction surgeries.

## Data Availability

The raw data supporting the conclusion of this article will be made available by the authors, without undue reservation.
